# Impact of timing, type, and intensity of physical activity on glycemic outcomes in a cohort of well-controlled youth with type 1 diabetes

**DOI:** 10.1007/s40618-025-02690-6

**Published:** 2025-09-30

**Authors:** Roberto Codella, Ambra Bisio, Marta Bassi, Nicola Minuto, Emilio Vichi, Daniel Gotti, Piero Ruggeri, Davide Maggi, Emanuela Faelli

**Affiliations:** 1 Department of Endocrinology, Nutrition and Metabolic Diseases, IRCCS MultiMedica, Milan, 20138 Italy; 2 Department of Biomedical Sciences for Health, Università degli Studi di Milano, Milan, 20133 Italy; 3 Centro Polifunzionale di Scienze Motorie, University of Genoa, Genoa, 16132 Italy; 4 Department of Experimental Medicine, Section of Human Physiology, University of Genoa, Genoa, 16132 Italy; 5 Pediatric Clinic, IRCCS Istituto Giannina Gaslini, University of Genoa, Genoa, Italy; 6 IRCCS San Martino, University of Genoa, Genoa, Italy

**Keywords:** Exercise, Physical activity, Type 1 diabetes, CGM, Glycemic control

## Abstract

**Aims:**

To evaluate how the timing, type, and intensity of bout-related physical activity (PA) influence glycemic control across age groups in youths and young adults with type 1 diabetes (T1D).

**Materials and methods:**

In this cross-sectional study, 100 insulin pump-treated individuals with T1D (55 females; mean age 16.6 ± 6.6 years; HbA1c 6.8 ± 1.0%) were monitored for 7 days using self-reported training logs and continuous glucose monitoring. Participants were categorized by age (< 14, 14–17, 18–31 years) and by exercise timing (morning, afternoon, evening), type (aerobic, anaerobic, mixed), and intensity (low, medium, high). Glycemic variables included mean glucose, time in range (TIR), time below range (TBR), time in level 2 hypoglycemia, time above range (TAR), time > 250 mg/dL, and total daily insulin dose.

**Results:**

Exercise timing and intensity had greater effects on glycemic outcomes than exercise type. Afternoon activity was associated with improved TIR and reduced TAR in younger participants, whereas morning exercise in adolescents was linked to higher TBR. Higher-intensity exercise was associated with greater TAR in adolescents whereas no significant differences in glycemic outcomes were found by exercise type.

**Conclusions:**

The timing and intensity of exercise significantly influence glycemic responses in youths with T1D, with notable age-related differences. Personalized PA recommendations should consider these factors to optimize glycemic outcomes.

**Supplementary Information:**

The online version contains supplementary material available at 10.1007/s40618-025-02690-6.

## Introduction

Physical activity (PA) plays a vital role in the management of type 1 diabetes (T1D), offering numerous health benefits such as improved cardiorespiratory fitness, lipid profiles, and a reduced long-term risk of cardiovascular disease [1–3]. Despite these benefits, PA in T1D presents unique challenges due to the lack of physiological insulin regulation. In particular, individuals with T1D must carefully adjust their insulin doses around exercise, as the risk of hypoglycemia − sometimes occurring even hours after PA − remains a significant concern [4, 5].

Hypoglycemia in the context of PA is largely attributable to several physiological factors. These include enhanced glucose absorption by active muscles, the inability of PA to sufficiently lower insulin levels in those using exogenous insulin, and heightened adrenergic activity that may exacerbate glucose fluctuations [1, 4–7]. Specifically, during exercise, individuals with T1D experience an increase in peripheral insulin sensitivity coupled with an impaired counter-regulatory hormonal response [8, 9]. This combination can create an imbalance between hepatic glucose production and muscular glucose utilization, potentially leading to exercise-induced hypoglycemia [10].

Given these complexities, optimizing the timing of PA may be essential to minimizing glycemic disturbances. While prior research has predominantly focused on exercise type and intensity, considerably less attention has been given to how the time of day may influence glycemic control in T1D. This knowledge gap is particularly relevant because circadian variations in hormonal and metabolic responses may affect glycemic outcomes during and after exercise [11].

Animal studies have provided some insight, indicating that the metabolic responses to exercise can vary depending on the time of day, with certain times potentially offering more favorable outcomes [12, 13]. In humans, limited studies suggest that morning exercise may be more effective in preventing late-onset hypoglycemia compared to afternoon exercise [14]. Exercising later in the day has been shown to cause a more pronounced decrease in blood glucose levels during PA and an increased risk of nocturnal hypoglycemia [15, 16]. Conversely, morning exercise sessions have been associated with a lower risk of hypoglycemia in the following 24 h and better overall glycemic time in range compared to evening or nighttime exercise sessions [14].

Further complicating the picture, the interaction between exercise type and timing may also influence glycemic outcomes. For instance, morning resistance training has been associated with transient hyperglycemia and increased glycemic variability, whereas afternoon resistance sessions may provide more stable post-exercise glucose profiles [17].

Despite these preliminary findings, the evidence remains inconsistent and insufficient, particularly in pediatric and young adult populations with T1D. To address this gap, the present study aims to evaluate how the timing, type, and intensity of unsupervised, bout-related PA affect glycemic outcomes in a real-world cohort of insulin pump–treated youths and young adults with T1D, monitored using flash continuous glucose monitoring. By examining these relationships across age groups, this study seeks to provide clearer guidance on the optimal structuring of PA to improve glycemic control in this population.

## Materials and methods

### Subjects

A total of one hundred outpatients with T1D were studied. The patients were recruited from a larger cohort of T1D patients attending follow-up visits at the Diabetes Clinic of the Gaslini and San Martino hospitals in Genoa, Italy. All participants were assessed for demographic, lifestyle, and anthropometric parameters. Participants’ body weight and height were measured to the nearest 0.1 kg and 1 mm, respectively, using a commercial scale/stadiometer device (Tanita BC 571, Tanita Corporation, Tokyo, Japan). BMI was calculated for each subject as weight (kg)/height (m)². Participants brought all prescription medications to the clinic visits to ensure accurate recording. Data were collected from March 2023 to September 2024.

**Inclusion criteria:** Patients older than 8 years of age with well-controlled T1D (CV, coefficient of variation < 36% in the 3 months prior to the commencement of the study), undergoing continuous glucose monitoring (CGM), and on continuous subcutaneous insulin infusion (CSII, insulin pump therapy) for at least 1 year.

**Exclusion criteria:** Pregnancy at the time of recruitment, presence of mental/psychological illnesses, use of beta blockers or glucocorticoids, peripheral vascular disease, peripheral neuropathy, and chronic kidney failure or dialysis therapy.

The protocol for this study was approved by the Ethics Committee of the University of Genoa (n° 23/2023). The study was conducted in accordance with the Helsinki Declaration of 1964 and its subsequent amendments. Informed consent was obtained from all participants or their legal guardians.

### Physical activity quantification and data grouping

In a cross-sectional design, subjects were screened for all types of PA performed over a seven-day period, in a fed state. The timing, duration, intensity, type, and volume of PA performed each day were recorded using a daily training log. The 7-day training recall is an established method for accurately capturing PA data in both healthy and diseased individuals, and is considered more reliable for assessing weekly PA volume than validated questionnaires [18]. At the time of the clinic appointment, patients were given a 7-day training log form, which contained detailed instructions to help them provide a precise description of their PA. Clinicians also provided patients with accurate instructions on how to correctly report data in the 7-day log. Each activity, with appropriate specifications on intensity, was converted into the corresponding metabolic equivalents of tasks per minute (METs • min) using the Ainsworth compendium, with separate references for adults and young people [19, 20]. Light-intensity activity was $\:\le\:\:$2.9 METs • min, moderate-intensity activity was comprised between 3–5.9 METs • min, high/vigorous-intensity was $\:\ge\:$ 6 METs • min. The Ainsworth compendium was likewise used to define the type/modality of PA/sports partaken [21]. The predominant time of day when subjects performed the highest volume of PA was assessed for each day of the week. If more than one activity was performed per day, differences greater than 10% in PA volume were used to categorize subjects as ‘morning’ (6 am − 12 pm), ‘afternoon’ (12 pm − 6 pm), or ‘evening/night’ (6 pm − 12 am) exercisers for that day. Daily data from all subjects were then grouped based on the predominant timing of exercise (morning, afternoon, or evening). Overnight bouts of exercise were excluded from the recordings.

### Glycemic variables

Continuous glucose changes were recorded using a FreeStyle Libre^®^ 3 (FSL, Abbott, CA, USA) device to assess glycemic variability throughout the day. Several glycemic variables were selected to represent glycemic variability and to describe episodes of hyperglycemia and hypoglycemia, including:

- Time in Range (TIR): 70–180 mg/dL.
- Time Below Range (TBR): <70 mg/dL.
- Time Below Extreme Hypoglycemia Range (TBER): <54 mg/dL.
- Time Above Range (TAR): 180–250 mg/dL.
- Time Above 250 mg/dL (TIME > 250): >250 mg/dL.
- Coefficient of Variation (CV) as a measure of glycemic variability.
- Mean Glucose.
- Total Daily Insulin Dose (TDD).

These variables were recorded daily over one week. Retrospective data downloaded from the CGM system could provide continuous glucose readings every 15 min. Daily glycemic data were matched with PA timepoints documented in the training log.

### Statistical analysis

Data are presented as mean ± standard error of the mean (SEM) except where differently indicated. The prevalence of type of physical activity/sports (aerobic, anaerobic, mixed), intensity (light, moderate, high), and preferred timing (morning, afternoon, night) among age group (under 14, 14–17, 18–31 years old) were assessed by exact tests like Chi square or Fisher’s, where appropriate. A two-way analysis of variance (ANOVA) was performed with age, type, intensity and timing as factors. The Greenhouse-Geisser correction was used when sphericity was not met (Mauchly test). Post hoc pair-wise comparisons were conducted utilizing Tukey’s test when main effect or interaction effects were demonstrated.

The significance level was set at 0.05 for all analyses.

Data analysis was performed using the Statistical Package for the Social Sciences (SPSS Statistics, version 29.0.2.0., IBM).

## Results

### Participants

The characteristics of the studied patients are reported in Table 1.

**Table 1:** Subjects’ characteristics

Youths with T1D (F/M)	55/45
Age (years) [min – max]	16 ± 6.6 [8–31]
BMI (kg/m^2^)	23 ± 3.4
Diabetes duration (years)	10 ± 5.8
CSII duration (years)	1.6 ± 0.18
HbA1c (%)	6.8 ± 1

Data are shown as mean ± SD

T1D: type 1 diabetes; BMI: body mass index; CSII: continuous subcutaneous insulin infusion; HbA1c: glycosylated hemoglobin

### Exercise timing, type, and intensity across age groups

**Fig. 1:**
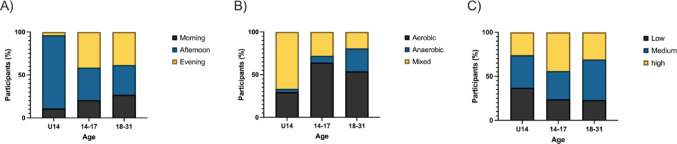
Exercise timing (**A**), type (**B**), and intensity (**C**) across age groups. Significant distribution in (**A**) with Chi square, and (**B**) with Fisher’s text

The distribution of exercise timing (Fig. 1A) revealed significant differences across age groups, with all groups showing a preference for a time of day (Chi-square = 18.54; df = 4, *p* = 0.001).

In terms of exercise type (Fig. 1B), participants under 14 years of age (U14) primarily engaged in mixed exercise, while groups of age 14–17 year and 18–31 years showed more balanced distributions of aerobic, anaerobic, and mixed modalities (Fisher’s, *p* = 0.0013).

For exercise load intensity (Fig. 1C), no significant differences were found in the distribution of low, medium, or high intensity training across age groups (Fisher’s, *p* > 0.05).

### Glycemic variables by exercise characteristics

**Fig. 2:**
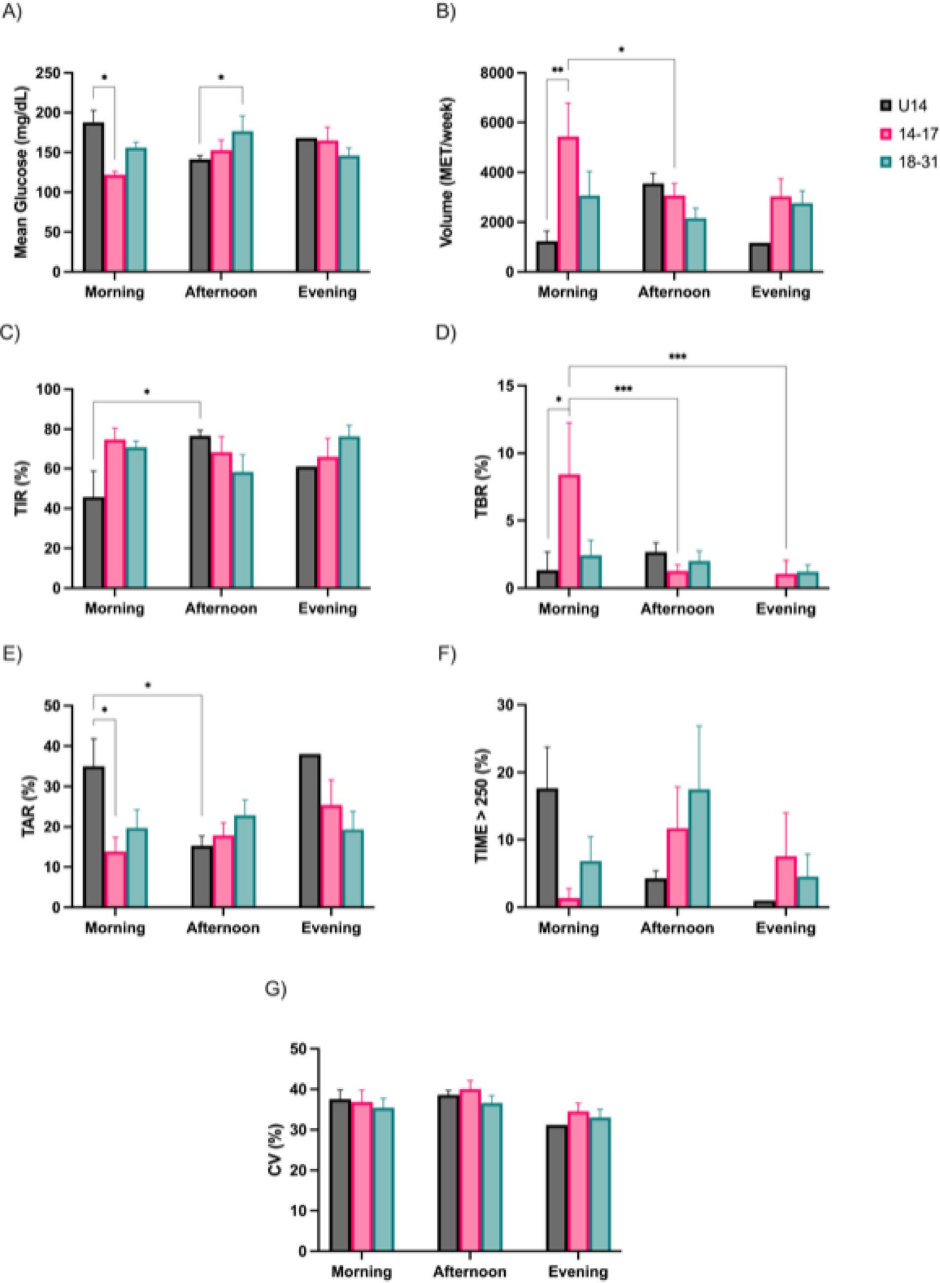
Glycemic variables and physical activity volume by exercise timing preference. Mean glucose (**A**); Volume of PA (**B**); Time in Range (**C**); Time Below Range (**D**); Time Above Range (**E**); Time Above 250 mg/dL (**F**). Coefficient of Variation (**G**) * *p* < 0.05; ** *p* < 0.01; *** *p* < 0.001

Significant variations in glycemic outcomes and exercise volume were observed according to the time of day and age group (Fig. 2). In morning sessions, the U14 group showed significantly higher mean glucose levels compared to adolescents aged 14–17 years (188 ± 15 vs. 122 ± 4.6 mg/dL, *p* < 0.05; Fig. 2A), and a similar difference was observed between the 18–31 and U14 age groups in the afternoon (177 ± 19.3 vs. 141 ± 4.7 mg/dL, *p* < 0.05; Fig. 2A). In the U14 group, TIR was significantly greater during afternoon exercise with respect to morning exercise (76.6 ± 2.8 vs. 45.7 ± 13.1%, *p* < 0.05; Fig. 2C). Correspondingly, TAR was elevated in the U14 group during morning compared to afternoon exercise (35 ± 11.8 vs. 15.3 ± 10.8%, *p* < 0.05; Fig. 2E). Also, during morning exercise, TAR was significantly greater in the U14 group with respect to 14–17 years (35 ± 6.8 vs. 13.8 ± 3.5%, *p* < 0.05; Fig. 2E).

Exercise volume (in MET/week) also varied significantly across time periods (Fig. 2B). Adolescents aged 14–17 years completed more morning training than the U14 group (5439 ± 1335 vs. 1228 ± 411 MET/week, *p* < 0.01; Fig. 2B). Moreover, adolescents aged 14–17 years exercised more in the morning with respect to afternoon (5439 ± 1335 vs. 3064 ± 486 MET/week, *p* < 0.05; Fig. 2B). Morning sessions were further characterized by higher Time Below Range (TBR) values in the 14–17 age group compared to younger participants (8.4 ± 3.8 vs. 1.3 ± 1.3%, *p* < 0.05, Fig. 2D). Also, in adolescents aged 14–17 years, morning TBR values significantly exceeded those recorded in afternoon and evening sessions (8.4 ± 3.8 vs. 1.3 ± 0.5 vs. 1.1 ± 1%, *p* < 0.001, Fig. 2D).

No statistically significant differences were observed in TIME > 250, although descriptive trends indicated a potential increase in the afternoon among young adults (Fig. 2F). CV values did not vary significantly across time periods (Fig. 2G).

**Fig. 3:**
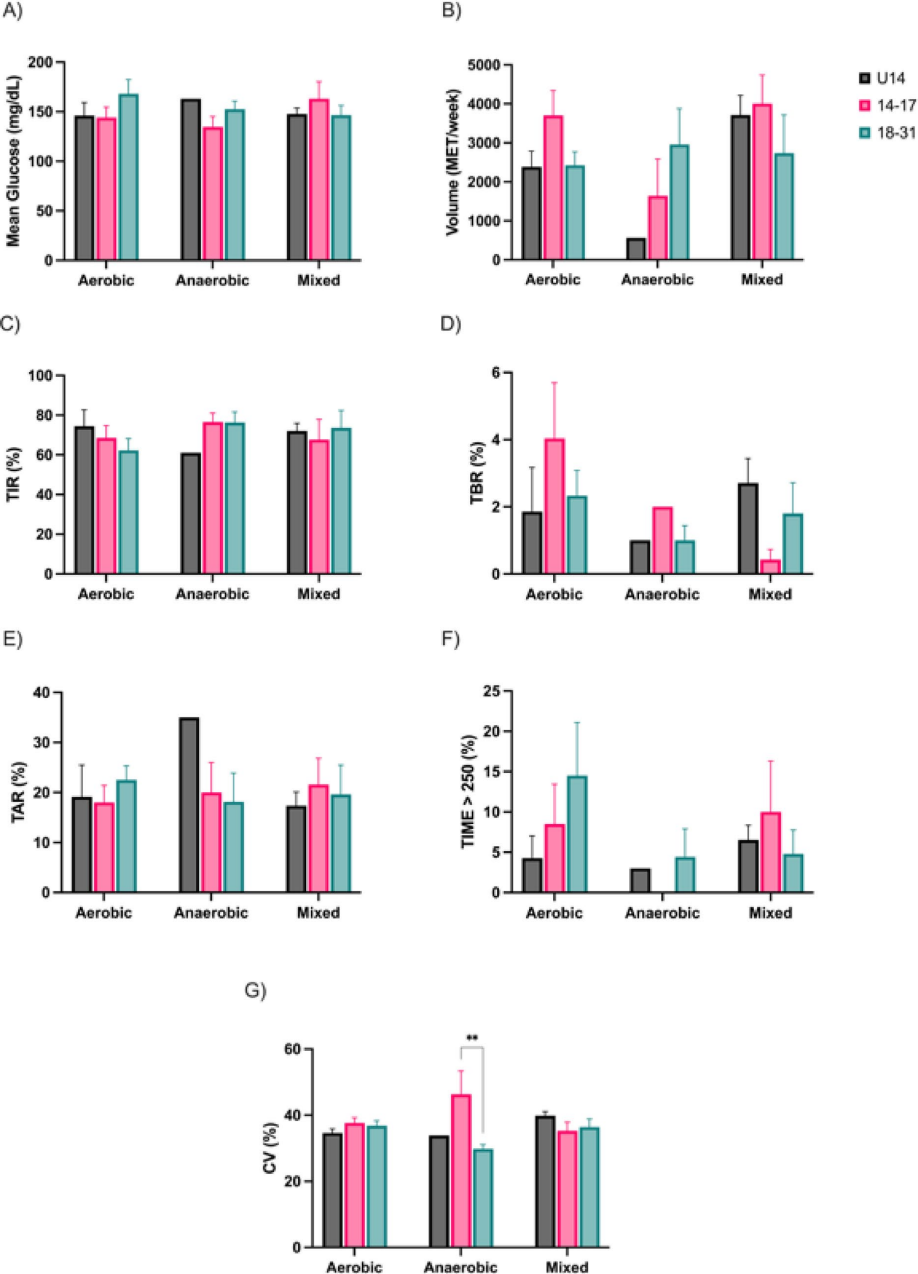
Glycemic variables and physical activity volume by type of exercise performed. Mean glucose (**A**); Volume of PA (**B**); Time in Range (**C**); Time Below Range (**D**); Time Above Range (**E**); Time Above 250 mg/dL (**F**). Coefficient of Variation (**G**)

When stratified by exercise type (aerobic, anaerobic, mixed), glycemic variables and training volumes displayed no statistically significant differences between age groups (Fig. 3A and B). Mean glucose, TIR, TBR, TAR, and time > 250 mg/dL remained comparable across conditions (Fig. 3C, D, E and F). Minor trends – such as slightly elevated TBR in aerobic sessions (Fig. 3D) and increased TAR in anaerobic training (Fig. 3E) among the adolescents aged 14–17 years – were noted but did not reach significance. Anaerobic exercise was associated with higher CV in the 14–17 group compared to the 18–31 group (46.5 ± 7 vs. 29.8 ± 0.7%, *p* < 0.01, Fig. 3G).

**Fig. 4:**
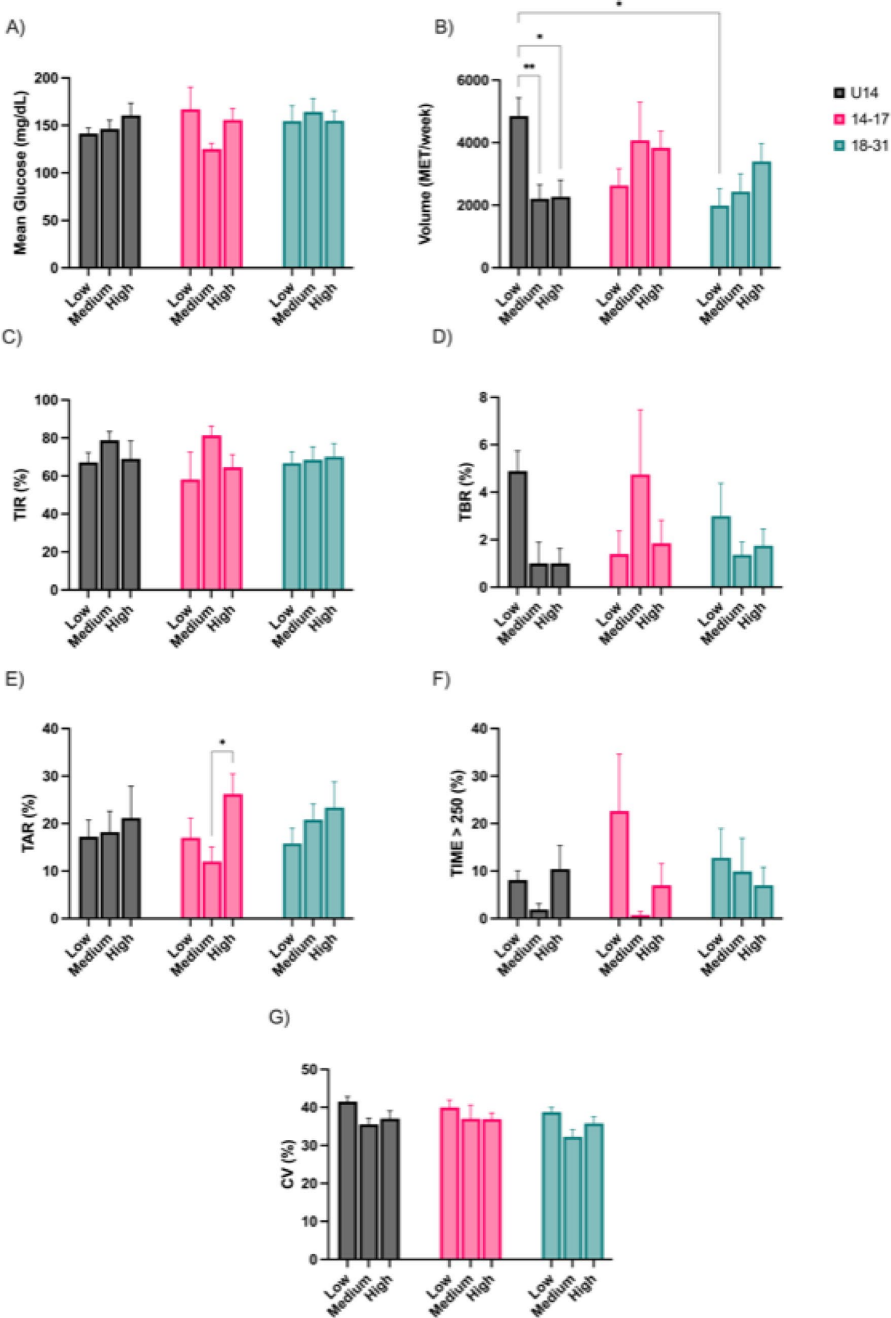
Glycemic variables and physical activity volume by intensity of exercise performed. Mean glucose (**A**); Volume of PA (**B**); Time in Range (**C**); Time Below Range (**D**); Time Above Range (**E**); Time Above 250 mg/dL (**F**). Coefficient of Variation (**G**) * *p* < 0.05; ** *p* < 0.01

When stratifying participants by training load intensity (low, medium, high), significant differences in exercise volume and selected glycemic variables emerged (Fig. 4). U14 participants exhibited significantly higher PA volume in the low-load condition compared to medium- (4853 ± 582 vs. 2207 ± 456 MET/week, *p* < 0.01; Fig. 4B) and high intensity category (4853 ± 582 vs. 2270 ± 538 MET/week, *p* < 0.05; Fig. 4B). Adolescents under 14 years showed higher volumes in low-load category compared to 18–31 age group (4853 ± 582 vs. 1989 ± 546 MET/week, *p* < 0.05; Fig. 4B).

Glycemic control showed modest variation across load levels. In the 14–17 group, TAR was significantly greater in the high-load condition compared to medium-load (26.2 ± 4.3 vs. 12 ± 3%, *p* < 0.05, Fig. 4E), indicating a possible post-exercise hyperglycemia.

No significant differences were observed in mean glucose (Fig. 4A), TIR (Fig. 4C), TBR (Fig. 4D), and TIME > 250 (Fig. 4F), across load conditions in any age group.

Glycemic responses in the young adult group (18–31 years) remained relatively stable across training intensities, with no statistically significant variations noted in any of the glucose variables. Similarly, no significant changes in CV were observed across training intensities (Fig. 4G).

We also conducted a gender analysis but did not find any statistically significant differences in glycemic responses (supplementary figure), exercise timing, type, or training intensity across the age groups.

## Discussion

In this cross-sectional single-center study, we investigated the associations between bout-related PA – specifically its timing, type, and intensity – and glycemic outcomes in youth and young adults with T1D. Our findings indicate that exercise timing and intensity are associated with select glycemic variables, whereas exercise type showed limited influence.

Differences in glycemic responses were observed across age groups and time-of-day subgroups. Adolescents aged 14–17 years demonstrated more favorable glycemic profiles during morning sessions, exhibiting significantly lower mean glucose compared to the younger groups (U14). Conversely, morning exercise in the U14 group was associated with higher TAR and lower TIR compared to afternoon exercise, suggesting that early-day PA may not yield optimal glycemic outcomes in this younger subgroup.

These findings echo prior studies indicating that morning exercise, particularly under fasted conditions, may contribute to greater glucose stability [14]. Yardley et al. [15], for example, reported smaller declines in blood glucose following fasted morning exercise compared to sessions performed later in the day. Nonetheless, such benefits may be age- and context-dependent, as our results highlight divergent responses across developmental stages. Notably, our study did not involve fasted training, which may partially explain why morning exercise was not universally beneficial – particularly among U14 participants.

Additionally, morning sessions in the 14–17 group were characterized by elevated TBR, both compared to their afternoon/evening sessions and to younger participants. This suggests that although mid-adolescents may experience improved glycemic control overall with morning PA, they could also face a higher risk of hypoglycemia, underscoring the need for vigilant glucose monitoring and individualized insulin/CHO adjustments. Physiologically, morning exercise occurs during a time of relatively low circulating insulin levels and heightened insulin sensitivity, particularly in adolescents, which can increase the risk of hypoglycemia. Additionally, the counter-regulatory hormonal response (e.g., cortisol, growth hormone) may be blunted in some individuals at this time of day. Behaviorally, adolescents may be less likely to adequately adjust insulin dosing or carbohydrate intake before morning physical activity, especially in unsupervised or routine school-day settings. These combined factors could contribute to an increased risk of post-exercise hypoglycemia in this group.

Contrary to earlier research suggesting distinct glycemic responses to aerobic versus anaerobic modalities [15–17, 22], our results showed no significant differences in glycemic variables across exercise types (aerobic, anaerobic, mixed). Mean glucose, TIR, TBR, TAR, and TIME > 250 mg/dL remained statistically comparable, with only minor non-significant trends observed.

These findings may be attributed to participants’ high proficiency in diabetes self-management tailored to activity demands. As such, exercise-triggered fluctuations may be attenuated when diabetes management is optimized.

Training intensity, on the other hand, was associated with significant differences in exercise volume, particularly among younger participants (U14). In contrast, adolescents aged 14–17 years exhibited higher TAR during high-load sessions, suggesting a risk of post-exercise hyperglycemia following more strenuous activity. These findings are in line with studies showing that high-intensity training or resistance workouts, particularly later in the day, can trigger acute glucose elevations due to counter-regulatory hormone release [15–17, 22].

Interestingly, glycemic variables in the 18-31-year group remained relatively stable across exercise intensities, implying that age-related metabolic factors or higher PA experience may buffer against glucose variability.

Several limitations should be acknowledged. First, our study did not assess immediate post-exercise glucose responses, precluding evaluation of acute hypoglycemia or rebound hyperglycemia. Second, nutritional strategies and caloric intake were not strictly controlled and they may represent potential confounders. Third, as all participants were youth and young adults, the findings may not be generalized to older adults, who often exhibit reduced counter-regulatory hormone responses, lower CHO oxidation, and impaired glycemic correction mechanisms [23, 24].

Moreover, participant chronotype was not measured. Previous research suggests that morning or evening preferences can impact exercise tolerance and glycemic responses [25]. This variable could partly explain the observed interindividual variability in glycemic outcomes based on time of day.

All participants had access to CGM and CSII and received regular training on insulin and CHO adjustments. This likely enhanced overall glycemic control and minimized exercise-related glycemic excursions, limiting the ability to detect larger between-condition differences.

Our findings underscore the need for future research to assess:

- Chronotype-exercise interactions in glycemic regulation;
- Immediate post-exercise glycemia, especially for high-risk periods like overnight;
- Longitudinal effects of routine PA timing and load on glycemic variability and HbA1c in broader populations, including older adults and those with suboptimal control.

Until such data are available, personalized exercise prescriptions remain critical. Rather than strictly enforcing exercise at a specific time, clinicians may achieve better adherence and outcomes by focusing on consistent, manageable PA routines tailored to individual glycemic patterns, preferences, and lifestyle factors.

While exercise timing and intensity influenced some glycemic variables in this well-managed cohort of young people with T1D, exercise type had minimal impact. Adolescents aged 14–17 years appeared to benefit metabolically from morning PA but also faced increased hypoglycemia risk. High-load training in adolescents were associated with glycemic variability, emphasizing the importance of age-specific, intensity-adjusted glycemic strategies. Overall, this study supports the promotion of regular PA in T1D, with a focus on individualization rather than rigid timing protocols.

## Supplementary Information

Below is the link to the electronic supplementary material.

Supplementary Material 1
