# Supplementary figures and images for: Impact of timing, type, and intensity of physical activity on glycemic outcomes in a cohort of well-controlled youth with type 1 diabetes
Source: J Endocrinol Invest. 2025 Sep 30;48(12):3003–12. doi: 10.1007/s40618-025-02690-6 (PMC12640312; doi:10.1007/s40618-025-02690-6)

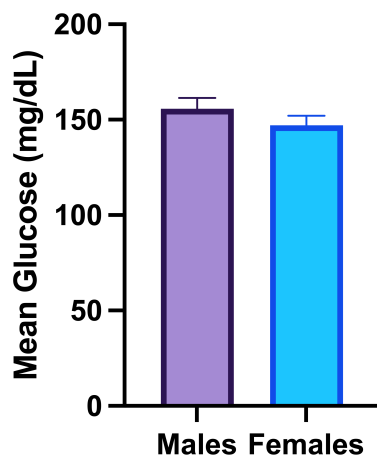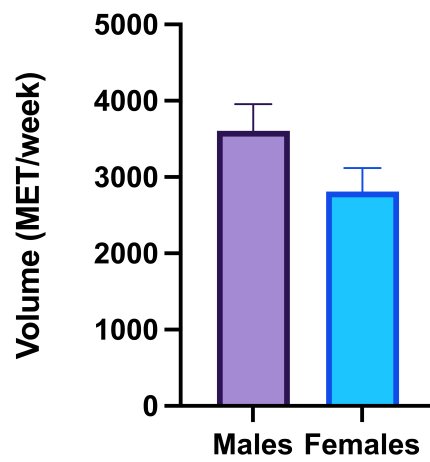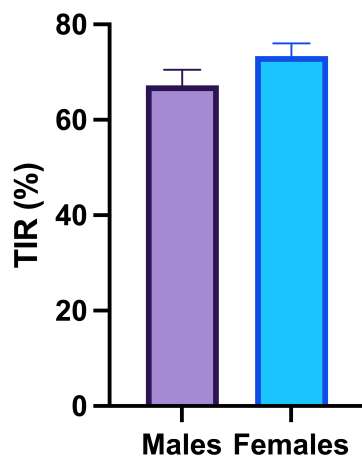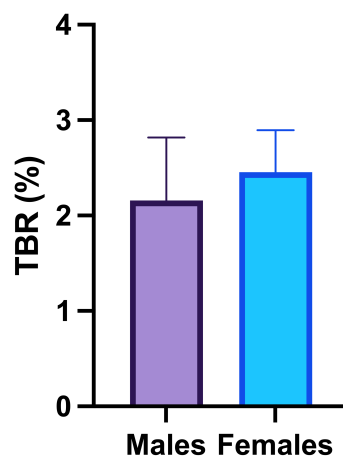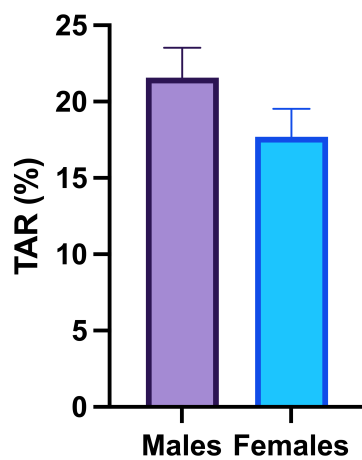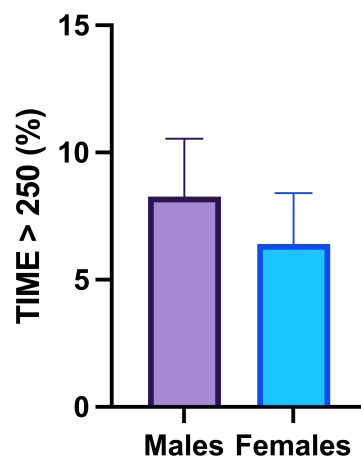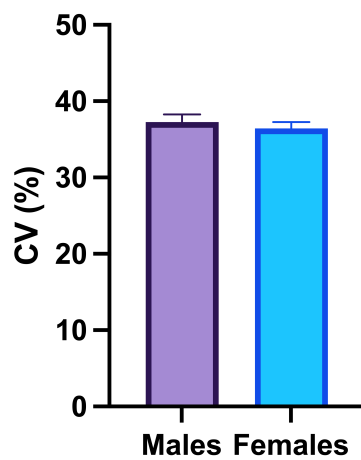

Supplement: Supplementary file 1 — Supplementary Material 1 [file 40618_2025_2690_MOESM1_ESM.pdf]
